# Supplementary material for: Dental microwear of a basal ankylosaurine dinosaur, Jinyunpelta and its implication on evolution of chewing mechanism in ankylosaurs
Source: PLoS One. 2021 Mar 10;16(3):e0247969. doi: 10.1371/journal.pone.0247969 (PMC7946176; doi:10.1371/journal.pone.0247969)

PLOS ONE

Dental microwear of a basal ankylosaurine dinosaur, Jinyunpelta and its implication on evolution of chewing mechanism in ankylosaurs.

--Manuscript Draft--

**Manuscript Number:**	PONE-D-20-40068
**Article Type:**	Research Article
**Full Title:**	Dental microwear of a basal ankylosaurine dinosaur, Jinyunpelta and its implication on evolution of chewing mechanism in ankylosaurs.
**Short Title:**	Dental microwear of a basal ankylosaurine dinosaur, Jinyunpelta.
**Corresponding Author:**	Tai Kubo<br>The University of Tokyo<br>Bunkyou-ku, Tokyo JAPAN
**Keywords:**	Dental microwear; Jinyunpelta; ankylosaurs; Cretaceous; jaw movement
**Abstract:**	The dental microwear of Jinyunpelta sinensis was observed using a confocal laser microscope. Jinyunpelta had steep wear facets that covered most of buccal surfaces of posterior dentary teeth. Observation of dental microwear on the wear facet revealed that scratch orientation varied according to its location within the wear facet: vertically (i.e. apicobasally) oriented scratches were dominant in upper half of the wear facet, and horizontally (i.e. mesiolaterally) oriented ones were in the bottom of the facet. These findings indicated that Jinyunpelta adopted precise tooth occlusion and biphasal jaw movement (orthal closure and palinal lower jaw movement). Among ankylosaurids, the biphasal jaw movement was previously only known from the Late Cretaceous North American taxa, but not for Asian ankylosaurs. The finding of biphasal jaw movement in Jinyunpelta showed sophisticate feeding adaptations emerged among ankylosaurids much earlier than previously thought. Parallel evolution of the biphasal jaw mechanism, which contemporaneously occurred among two lineages of ankylosaurs, ankylosaurids and nodosaurids, might reflect changes in paleoflora during the late Early to the early Late Cretaceous.
**Order of Authors:**	Tai Kubo
	Wenjie Zheng
	Xingsheng Jin
	Mugino Ozaki Kubo
**Additional Information:**	
**Question**	**Response**
**Financial Disclosure**<br><br>Enter a financial disclosure statement that describes the sources of funding for the work included in this submission. Review the submission guidelines for detailed requirements. View published research articles from *PLOS ONE* for specific examples.<br><br>This statement is required for submission and **will appear in the published article** if the submission is accepted. Please make sure it is accurate.	TK was funded by JSPS KAKENHI grant number 19J40003. WZ and XJ were funded by Chinese Natural Science Foundation (41602019). The funders had no role in study design, data collection and analysis, decision to publish, or preparation of the manuscript.

Powered by Editorial Manager® and ProduXion Manager® from Aries Systems Corporation

**Unfunded studies**<br>Enter: *The author(s) received no specific funding for this work.*<br><br>**Funded studies**<br>Enter a statement with the following details:<br>• Initials of the authors who received each award<br>• Grant numbers awarded to each author<br>• The full name of each funder<br>• URL of each funder website<br>• Did the sponsors or funders play any role in the study design, data collection and analysis, decision to publish, or preparation of the manuscript?<br>• **NO** - Include this sentence at the end of your statement: *The funders had no role in study design, data collection and analysis, decision to publish, or preparation of the manuscript.*<br>• **YES** - Specify the role(s) played.<br><br>* typeset	
**Competing Interests**<br><br>Use the instructions below to enter a competing interest statement for this submission. On behalf of all authors, disclose any competing interests that could be perceived to bias this work—acknowledging all financial support and any other relevant financial or non-financial competing interests.<br><br>This statement **will appear in the published article** if the submission is accepted. Please make sure it is accurate. View published research articles from *PLOS ONE* for specific examples.	The authors have declared that no competing interests exist.

**NO authors have competing interests**<br><br>Enter: *The authors have declared that no competing interests exist.*<br><br>**Authors with competing interests**<br><br>Enter competing interest details beginning with this statement:<br><br>*I have read the journal's policy and the authors of this manuscript have the following competing interests: [insert competing interests here]*<br><br>* typeset	
**Ethics Statement**<br><br>Enter an ethics statement for this submission. This statement is required if the study involved:<br><br>• Human participants<br>• Human specimens or tissue<br>• Vertebrate animals or cephalopods<br>• Vertebrate embryos or tissues<br>• Field research<br><br>Write "N/A" if the submission does not require an ethics statement.<br><br>General guidance is provided below. Consult the submission guidelines for detailed instructions. **Make sure that all information entered here is included in the Methods section of the manuscript.**	N/A

**Format for specific study types**<br><br>**Human Subject Research (involving human participants and/or tissue)**<br>• Give the name of the institutional review board or ethics committee that approved the study<br>• Include the approval number and/or a statement indicating approval of this research<br>• Indicate the form of consent obtained (written/oral) or the reason that consent was not obtained (e.g. the data were analyzed anonymously)<br><br>**Animal Research (involving vertebrate animals, embryos or tissues)**<br>• Provide the name of the Institutional Animal Care and Use Committee (IACUC) or other relevant ethics board that reviewed the study protocol, and indicate whether they approved this research or granted a formal waiver of ethical approval<br>• Include an approval number if one was obtained<br>• If the study involved *non-human primates*, add *additional details* about animal welfare and steps taken to ameliorate suffering<br>• If anesthesia, euthanasia, or any kind of animal sacrifice is part of the study, include briefly which substances and/or methods were applied<br><br>**Field Research**<br><br>Include the following details if this study involves the collection of plant, animal, or other materials from a natural setting:<br>• Field permit number<br>• Name of the institution or relevant body that granted permission	
**Data Availability**<br><br>Authors are required to make all data underlying the findings described fully available, without restriction, and from the time of publication. PLOS allows rare exceptions to address legal and ethical concerns. See the PLOS Data Policy and FAQ for detailed information.	Yes - all data are fully available without restriction

A Data Availability Statement describing where the data can be found is required at submission. Your answers to this question constitute the Data Availability Statement and **will be published in the article**, if accepted.<br><br>**Important:** Stating 'data available on request from the author' is not sufficient. If your data are only available upon request, select 'No' for the first question and explain your exceptional situation in the text box.<br><br>Do the authors confirm that all data underlying the findings described in their manuscript are fully available without restriction?	
**Describe where the data may be found in full sentences. If you are copying our sample text, replace any instances of XXX with the appropriate details.**<br><br>• If the data are **held or will be held in a public repository**, include URLs, accession numbers or DOIs. If this information will only be available after acceptance, indicate this by ticking the box below. For example: *All XXX files are available from the XXX database (accession number(s) XXX, XXX.*).<br>• If the data are all contained **within the manuscript and/or Supporting Information files**, enter the following: *All relevant data are within the manuscript and its Supporting Information files.*<br>• If neither of these applies but you are able to provide **details of access elsewhere**, with or without limitations, please do so. For example:<br><br>*Data cannot be shared publicly because of [XXX]. Data are available from the XXX Institutional Data Access / Ethics Committee (contact via XXX) for researchers who meet the criteria for access to confidential data.*<br><br>*The data underlying the results presented in the study are available from (include the name of the third party*	All relevant data are within the manuscript and its Supporting Information files.

and contact information or URL).<br>• This text is appropriate if the data are owned by a third party and authors do not have permission to share the data.<br><br>* typeset	
Additional data availability information:	

main text Click here to access/download;Manuscript;manusrcipt20201221.docx

Dental microwear of a basal ankylosaurine dinosaur, *Jinyunpelta* and its implication on evolution of chewing mechanism in ankylosaurs.

Tai Kubo[1,*], Wenjie Zheng[2], Mugino O. Kubo[3], Xingsheng Jin[2]

[1] The University Museum, The University of Tokyo, Tokyo, Japan

[2] Zhejiang Museum of Natural History, Hangzhou, Zhejiang, People's Republic of China

[3] Department of Natural Environmental Studies, Graduate School of Frontier Sciences, The University of Tokyo, Kashiwa, Chiba, Japan

* Corresponding author

E-mail: taikubo@um.u-tokyo.ac.jp

Abstract

The dental microwear of *Jinyunpelta sinensis* was observed using a confocal laser microscope. *Jinyunpelta* had steep wear facets that covered most of buccal surfaces of posterior dentary teeth. Observation of dental microwear on the wear facet revealed that scratch orientation varied according to its location within the wear facet: vertically (i.e. apicobasally) oriented scratches were dominant in upper half of the wear facet, and horizontally (i.e. mesiolaterally) oriented ones were in the bottom of the facet. These findings indicated that *Jinyunpelta* adopted precise tooth occlusion and biphasal jaw movement (orthal closure and palinal lower jaw movement). Among ankylosaurids, the biphasal jaw movement was previously only known from the Late Cretaceous North American taxa, but not for Asian ankylosaurs. The finding of biphasal jaw movement in *Jinyunpelta* showed sophisticate feeding adaptations emerged among ankylosaurids much earlier than previously thought. Parallel evolution of the biphasal jaw mechanism, which contemporaneously occurred among two lineages of ankylosaurs, ankylosaurids and nodosaurids, might reflect changes in paleoflora during the late Early to the early Late Cretaceous.

Introduction

Ankylosaurs were herbivorous dinosaurs that emerged in the Middle Jurassic and prospered until the end of Cretaceous [1,2]. During the Late Cretaceous, compared to other contemporaneous

megaherbivores, such as ceratopsians and hadrosaurs, ankylosaurs likely fed on less fibrous plants growing in lower layers of paleovegetation [3,4]. Ankylosaurs were assumed to feed mainly on herbaceous ferns [4], which were dominant in stomach contents of an Early Cretaceous nodosaurid ankylosaur, *Borealopelta*, implying that this animal fed on ferns selectively [5]. Stomach contents of another Early Cretaceous basal ankylosaur, *Kunbarrasaurus*, also contained possible fern sporangia along with vascular tissue, angiosperm fruits, and small seeds [6,7]. Among two main ankylosaurian families, i.e. Nodosauridae and Ankylosauridae, the former was assumed to have consumed more fibrous and tougher food than the latter, but dental microwear of Late Cretaceous North American taxa did not show distinction between these two families [3]. Not only diets but also jaw mechanism were implicated in ankylosaur dental microwear. Contrary to the expectation that ankylosaurs adopted simple orthal jaw movement due to their small and simple leaf-shaped teeth, dental microwear of derived Late Cretaceous taxa, such as an ankylosaurid *Euoplocephalus* [8] and nodosaurids, *Panoplosaurus* and *Hungarosaurus*, [3,9] indicated biphasal jaw mechanism that adopted both orthal closure and palinal lower jaw movements.

It has been shown by analyses of skull morphometrics and dental wear that ankylosaurs evolved the biphasal jaw mechanism convergently in different ankylosaurian lineages [10]. According to Ősi et al. [10], basal ankylosaurs used orthal jaw movement, during which teeth rarely occluded. Precise tooth occlusion evolved convergently among basal nodosaurids and at the common ancestor of derived

North American ankylosaurids. Within these groups with tooth occlusion, palinal jaw movement evolved convergently several times. An Early Cretaceous nodosaurid *Sauropelta* might have been adopted palinal jaw movements. This would be also the cases for Late Cretaceous nodosaurids, *Struthiosaurus* and *Panoplosaurus* as well as for a Late Cretaceous ankylosaurid *Ankylosaurus*. On the other hand, there were clear evidences of palinal jaw movements for Late Cretaceous nodosaurids *Edmontonia*, *Panoplosaurus*, *Hungarosaurus*, and a Late Cretaceous ankylosaurid *Euoplocephalus* [10]. Asian ankylosaurids, however, were thought to retain ancestral orthal jaw movement without tooth occlusion until the end of Cretaceous [10]. The evolutionary pattern of jaw mechanism in ankylosaurs is more complex than those of other herbivorous dinosaur taxa. For example, hadrosaurids exhibited a continuous evolutionary trend toward a more efficient masticatory system throughout Cretaceous, such as an increase of functional teeth and invention of new dental tissue types [11,12]. Since ankylosaurs exhibited convergent evolution in their feeding mechanism more frequently than other herbivorous dinosaurs, more species needed to be examined for ankylosaurs to reconstruct the feeding mechanism evolution. Also, to deduce the factors that promoted the convergent evolution of sophisticated feeding adaptations in different ankylosaurian lineages, data from ankylosaur species that lived in various regions and periods were demanded.

Jinyunpelta sinensis is the most basal ankylosaurine and the oldest ankylosaur with a tail club, which was excavated by a joint team of Zhejiang Museum of Natural History (ZMNH), Jinyun

Museum, and Fukui Prefectural Dinosaur Museum during 2013 from Albian–Cenomanian Liangtoutang Formation of Jinyun County, Zhejiang Province, China [13]. During the excavation, more than five individuals were found from the construction site. The subsequent description was based on two specimens (Holotype ZMNH M8960 and Paratype ZMNH M8963) as other specimens were still under preparation [13]. The holotype skull ZMNH M8960 is dorsoventrally compressed and its upper and lower teeth rows are tightly occluded, therefore existence or absence of wear facet(s) on teeth could not be determined. Lately, it was clarified through preparation that another *Jinyunpelta* specimen ZMNH M8961 conserved the posterior part of dentaries and maxillae (Fig 1). Due to postmortem damages, most teeth were fallen out from teeth sockets and surfaces of many *in situ* teeth were abraded, but several posterior-most teeth of the right dentary were untouched exposing their buccal sides. Also, one left dentary tooth exposed undamaged lingual surface (Fig 1f). Further, a few teeth of the right dentary that positioned more apical (dorsal) to adjacent teeth, which were probably functional when the animal lived, exhibited steeply inclined wear facets on their buccal sides (Fig 1b). This contradicts with the notion of Ősi et al. [10] that Asian ankylosaurids rarely showed low-angled apical wear, implying the absence of precise tooth occlusion. In this study, therefore, we observed the dental microwear of *Jinyunpelta* to deduce its jaw movement and to reconstruct the evolution of the feeding mechanism in Asian and Cretaceous ankylosaurids.

Fig 1. Dental microwear of *Jinyunepelta*. (a) Right lateral view of the skull of *Jinyunpelta* ZMNH M8961. (b) close up view of buccal sides of the right dentary teeth A, B, and C. (c) Dental microwear at the border between wear facet and non-wear facet on the tooth A. (d) Dental microwear of the wear facet on the tooth B. (e) Dental microwear of the buccal surface of the tooth C. (f) Close up of the lingual side of the left dentary showing the tooth D (g) Dental microwear of the lingual surface of the tooth D. All photosimularions were taken from molds using a confocal laser microscope (VK-9700) with either a 100× or 10× objective lens. The field of view is 140×105 μm for 100× lens and 1403×1052 μm for 10× lens. Photosimularions were mirrored in the horizontal direction to match its direction with the real tooth surfaces. Abbreviations: den, dentary; l, left; mx, maxilla; or, orbit; r, right; wf, wear facet.

Materials and Methods

Molding procedures were following the methodology of Kubo and Kubo [14]. Teeth of ZMNH M8961 that preserved shiny surfaces with none or minor visible damages were selected and cleaned by cotton swabs soaked in acetone. After the cleaning, molds were taken using high-resolution A-silicone dental impression material (Dr. Silicon regular type, DentKist, Inc., Korea). Buccal surfaces of six right dentary teeth that include two wear facets and one possible wear facet (Fig 1b) and one lingual surface of the left dentary tooth (Fig 1f) were molded.

Preservation of dental impressions was observed using a confocal laser microscope (VK-9700, Keyence, Osaka, Japan) and when the preservation of the entire visual field was satisfactory, the dental impressions were scanned using a violet laser (wavelength 408nm) with 10× and long-distance 100× objective lenses (numerical apertures 0.30 and 0.95 respectively). For a long-distance 100× objective lens, the scan pitches were 0.138 μm/pixel for x- and y- axes, and for a 10× objective lens that are 1.379 μm/pixel. A vertical resolution was 1 nm for both lenses. By scanning, the light intensity of reflected laser, color information, and height data (Z position) were obtained for each XY coordinate point at each focus point. Using these data, VK-9700 generates real color ultra-depth images (photosimulation), from which dental microwear were visually checked. Each photosimulation was 1024×768 pixel flame, therefore covering the area of 141×106 μm with long-distance 100× objective lens and 1412×1059 μm with 10× objective lens.

Scratch orientation differed apicobasally within the same wear facet in some ankylosaurs, reflecting a multiphasic, complex jaw mechanism [9]. Therefore, we scanned wear facets in apicobasal direction using 10× objective lens, to make the image of the entire apicobasal section by combining photosimulation images.

Results

By examining photosimulated images of mold surfaces, microwear was preserved in four teeth,

which hereafter referred as tooth A, B, C, and D (Fig. 1): buccal wear facets of two right dentary teeth (teeth A and B), the buccal non-wear facet surface of the right dentary tooth (tooth C), and the lingual non-wear facet surface of the left dentary tooth (tooth D). These teeth surfaces must have been affected by taphonomic processes to some extent as cracks were observed on the surface of the wear facet of the tooth B (Fig 1d). Taphonomic processes, however, tend to obliterate rather than create dental microwear [15], thus the observed orientation and density of scratches were unlikely to be affected by taphonomy.

The density of scratches was low in non-wear facets and high in wear facets (Fig 1). The dental microwear of wear facet was probably produced by attrition (tooth-tooth contact) and that of the non-wear facet was made by abrasion (tooth-food contact). Therefore, this difference in microwear densities meets the expectation and further confirming that observed scratches were not extensively affected by the postmortem taphonomic process. Directions of scratches were more aligned on wear facets compared with non-wear facets (Fig1). The orientation of scratches on wear facets changes between apicobasal positions within wear facets of teeth A and B. This orientation change can be observed clearer in the wear facet of tooth B compared with that of tooth A, because of its better preservation throughout the apicobasal axis (Fig 2). In the wear facet of tooth B, at the apical region, scratches were inclined mesially (anteriorly) about 30 degrees from the apicobasal axis. In the middle region, scratches of different orientations crossed each other; the ones that inclined mesially

(anteriorly) about 20 degrees from the apicobasal axis and another one that inclined distally (posteriorly) about 50 degrees from the apicobasal axis. At the basal regions, almost all scratches were inclined distally (posteriorly) about 60 degrees from the apicobasal axis (Fig 2). In the wear facet of tooth A, scratches were inclined mesially (anteriorly) about 30 degrees from the apicobasal axis at the apical area of the wear facet (Fig 1c). At the basal half of the wear facet, preservation of microwear was not as good as the apical half, but it seemed scratches of two orientations coexist, one that inclined mesially (anteriorly) about 30 degrees from the apicobasal axis and another one that inclined distally (posteriorly) about 60 degrees from the apicobasal axis.

Fig 2. Changes in the orientation of scratches along the apicobasal axis of wear facet. (a) The buccal wear facet of the tooth B. (b) Dental microwear along the entire apicobasal axis of the wear facet that is enclosed by the black rectangle in Fig 2a. Scratches are emphasized by white lines. The width of the image is 1.4 mm. (c) The close up of the middle region of wear facet, where scratch orientations change apicobasally and is enclosed by the white rectangle in Fig 2b. The width of the image is 1.4 mm.

Discussion and Conclusions

Scratch orientation found in wear facets of *Jinyunpelta* teeth changed from less mesially inclined

ones at the apical position of wear facet and more inclined ones at the basal position, that is almost identical to that of a nodosaurid ankylosaur *Hungarosaurus* [9: Fig. 6]. Therefore, the chewing jaw movement of these two taxa must be similar. The orientation of scratches, which inclines 20 to 30 degrees mesially from the apicobasal axis in the apical region of the wear facet indicates teeth occlusion start with orthal movement in *Jinyunpelta*. The existence of two scratch orientations that cross each other at the middle region of wear facet might reflect an abrupt shift of lower jaw movement from orthal to the backward and slightly upward direction. During this backward traction, the apical region of wear facets did not have contact with the antagonist tooth. The dominance of the low-angled, mesiobasal-apicodistal oriented scratches at the basal region of the wear facet indicates that the chewing cycle ended with the movement of lower jaw in the backward and slightly upward direction.

As stated by Mallon and Anderson [3] “The microwear signal in one area of a tooth often differed from that in another area of the same tooth”. In some dinosaurian taxa, the area within the wear facet that occluded with the antagonist tooth changes with the phase of chewing cycle. Therefore, if directions of jaw movements change along with the phase of the chewing cycle (e.g. *Leptoceratops* and ankylosaurs with biphasal jaw movement), the orientation of dental microwear differs in different areas of wear facet [9,16]. Therefore, preferably, observation of the entire apicobasal axis within a wear facet is needed to reconstruct the jaw movement of the entire chewing cycle. However, this is practically impossible in many cases as the preservation of whole wear facet is rarely satisfactory to

analyze dental microwear.

The dental microwear feature of *Jinyunpelta* exhibited clear similarity with that of *Hungarosaurus* and *Euoplocephalus* in having long and aligned scratches [10]. These taxa, including *Jinyunpelta*, also resemble in their chewing manner that adopted biphasal jaw movement. Scratches of *Jinyunpelta* are much denser than other ankylosaurs that fills the entire field of view and are almost countless. ~~The specimen of~~ an isolated ankylosaurid tooth (TMP 1991.050.0014) that was observed in Mallon and Anderson [3: Fig. 1] is probably the sole example that is comparable in the density of scratches. The variance in microwear parameters (i.e. numbers and widths of pits and scratches) is larger in ankylosaurs compared with other herbivorous dinosaurs likely due to their imprecise tooth occlusion [3]. Nonetheless, the above similarities and differences between dental microwear of *Jinyunpelta* and other ankylosaurs likely correspond to similarities and differences in their diets and chewing styles.

Among ankylosaurs, precise tooth occlusion was more widely seen among nodosaurids compared with ankylosaurids. Among ankylosaurids, the precise tooth occlusion was previously known only for the Late Cretaceous North American taxa (Fig 3) and the definite evidence of palinal jaw movement was known only for *Euoplocephalus* [8,10]. The precise tooth occlusion with palinal jaw movement of *Jinyunpelta* confirmed in this study, however, indicate sophisticated chewing jaw movement evolved much earlier among ankylosaurids, in the late Early or early Late Cretaceous (Fig. 3).

Fig 3. Evolution of chewing mechanism in ankylosaurs. The color of each genus name represents the continent they lived: red, Asia; orange, North America; green, Europe. Modified from Ősi et al. [10: Fig. 15]. The phylogenetic relationship of ankylosaurs followed [13].

This study is the first to show the solid evidence of precise tooth occlusion for Asian ankylosaurs. Asian ankylosaurs had been known to exhibit none or small nearly horizontal apical wear facets [10]. Therefore, steep wear facets of dentary teeth of *Jinyunpelta* that cover the most area of buccal surface is unique among Asian ankylosaurs. The lack of sophisticated feeding adaptations in Asian ankylosaurs and its presence in Late Cretaceous Ankylosaurs of Europe and North America, was suspected to have been reflecting habitat differences among different continents [10]. The finding that contemporaneous Asian ankylosaurs, *Jinyunpelta* and *Gobisaurus*, differ in their chewing systems, indicated that these differences reflect local habitat differences and/or dietary differences among closely related taxa. The findings of tooth occlusion and biphasal jaw mechanism in *Jinyunpelta* that is phylogenetically nested within Asian taxa without tooth occlusion indicates the evolution of a complex feeding mechanism could be evolved easily among ankylosaurs. Roughly contemporaneous emergence of palinal jaw movement within both ankylosaurids and nodosaurids during the late Early to the early Late Cretaceous (Fig 3) corroborates the idea that these functional novelties of ankylosaurs jaw movement were triggered by changes in paleoflora [10].

Acknowledgments

We thank the following people for participating the fieldwork in 2013: S. Gu (ZMNH), C. Chen (Jinyun Museum), K. Miyata (Fukui Prefectural Dinosaur Museum), and several local farmers. We thank C. Yu, A. Liu and Y. Sheng (ZMNH) for preparing the specimens and H. Sakaki (University of Tokyo) for help in molding dental impressions at ZMNH.

References

1. Arbour VM, Currie PJ. Systematics, phylogeny and palaeobiogeography of the ankylosaurid dinosaurs. J Syst Palaeontol. 2016;14: 385–444. doi:10.1080/14772019.2015.1059985
2. Mallon JC, Evans DC, Ryan MJ, Anderson JS. Feeding height stratification among the herbivorous dinosaurs from the Dinosaur Park Formation (upper Campanian) of Alberta, Canada. BMC Ecol. 2013;13: 14. doi:10.1186/1472-6785-13-14
3. Mallon JC, Anderson JS. The Functional and Palaeoecological Implications of Tooth Morphology and Wear for the Megaherbivorous Dinosaurs from the Dinosaur Park Formation (Upper Campanian) of Alberta, Canada. PLoS One. 2014;9: e98605. doi:10.1371/journal.pone.0098605
4. Mallon JC. Competition structured a Late Cretaceous megaherbivorous dinosaur assemblage. Sci Rep. 2019;9: 1–18. doi:10.1038/s41598-019-51709-5

5. Brown CM, Greenwood DR, Kalyniuk JE, Braman DR, Henderson DM, Greenwood CL, et al. Dietary palaeoecology of an Early Cretaceous armoured dinosaur (Ornithischia; Nodosauridae) based on floral analysis of stomach contents. R Soc Open Sci. 2020. doi:10.1098/rsos.200305

6. Molnar RE, Clifford TH. An Ankylosaurian Cololite from the Lower Cretaceous of Queensland, Australia. In: Carpenter K, editor. The armored dinosaurs. Indiana University Press; 2001. pp. 399–412.

7. Molnar RE, Clifford TH. Gut contents of a small ankylosaur. J Vertebr Paleontol. 2000;20: 194–196.

8. Rybczynski R, Vickaryous MK. Evidence of complex jaw movement in the Late Cretaceous ankylosaurid *Euoplocephalus tutus*. In: Carpenter K, editor. The Armored Dinosaurs. Bloomington: Indiana University Press; 2001. pp. 299–317.

9. Ősi A, Barrett PM, Földes T, Tokai R. Wear Pattern, Dental Function, and Jaw Mechanism in the Late Cretaceous Ankylosaur *Hungarosaurus*. Anat Rec. 2014. doi:10.1002/ar.22776

10. Ősi A, Prondvai E, Mallon J, Bodor ER. Diversity and convergences in the evolution of feeding adaptations in ankylosaurs (Dinosauria: Ornithischia). Hist Biol. 2017;29: 539–570. doi:10.1080/08912963.2016.1208194

11. Suarez CA, You HL, Suarez MB, Li DQ, Trieschmann JB. Stable Isotopes Reveal Rapid Enamel Elongation (Amelogenesis) Rates for the Early Cretaceous Iguanodontian Dinosaur

Lanzhousaurus magnidens. Sci Rep. 2017;7: 1–8. doi:10.1038/s41598-017-15653-6

12. Erickson GM, Krick BA, Hamilton M, Bourne GR, Norell MA, Lilleodden E, et al. Complex dental structure and wear biomechanics in hadrosaurid dinosaurs. Science. 2012;338: 98–101. doi:10.1126/science.1224495

13. Zheng W, Jin X, Azuma Y, Wang Q, Miyata K, Xu X. The most basal ankylosaurine dinosaur from the Albian-Cenomanian of China, with implications for the evolution of the tail club. Sci Rep. 2018;8: 3711. doi:10.1038/s41598-018-21924-7

14. Kubo T, Kubo MO. Dental microwear of a Late Triassic dinosauriform, *Silesaurus opolensis*. Acta Palaeontol Pol. 2014;59: 305–312. doi:10.4202/app.2013.0027

15. King T, Andrews P, Boz B. Effect of taphonomic processes on dental microwear. Am J Phys Anthropol. 1999;108: 359–373. doi:10.1002/(SICI)1096-8644(199903)108:3<359::AID-AJPA10>3.0.CO;2-9

16. Varriale FJ. Dental microwear reveals mammal-like chewing in the neoceratopsian dinosaur *Leptoceratops gracilis*. PeerJ. 2016;4: e2132. doi:10.7717/peerj.2132

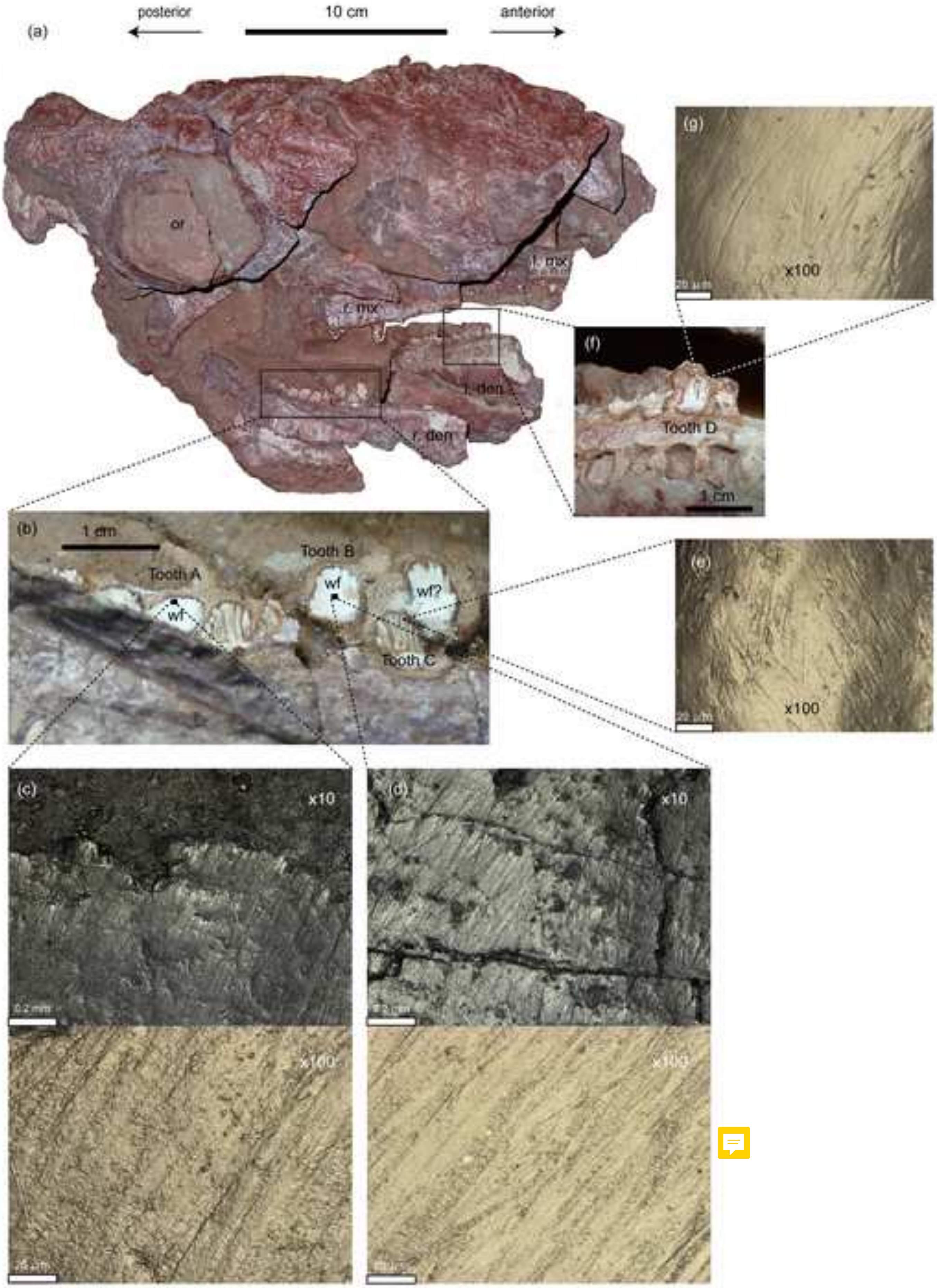

(a)
posterior
10 cm
anterior
or
l. mx
r. mx
l. den
r. den
(b)
1 cm
Tooth A
Tooth B
Tooth C
wf
wf
wf?
(c)
x10
x100
(d)
x10
x100
(e)
x100
(f)
Tooth D
1 cm
(g)
x100

Figure 2

Click here to access/download;Figure;Fig 2.jpg

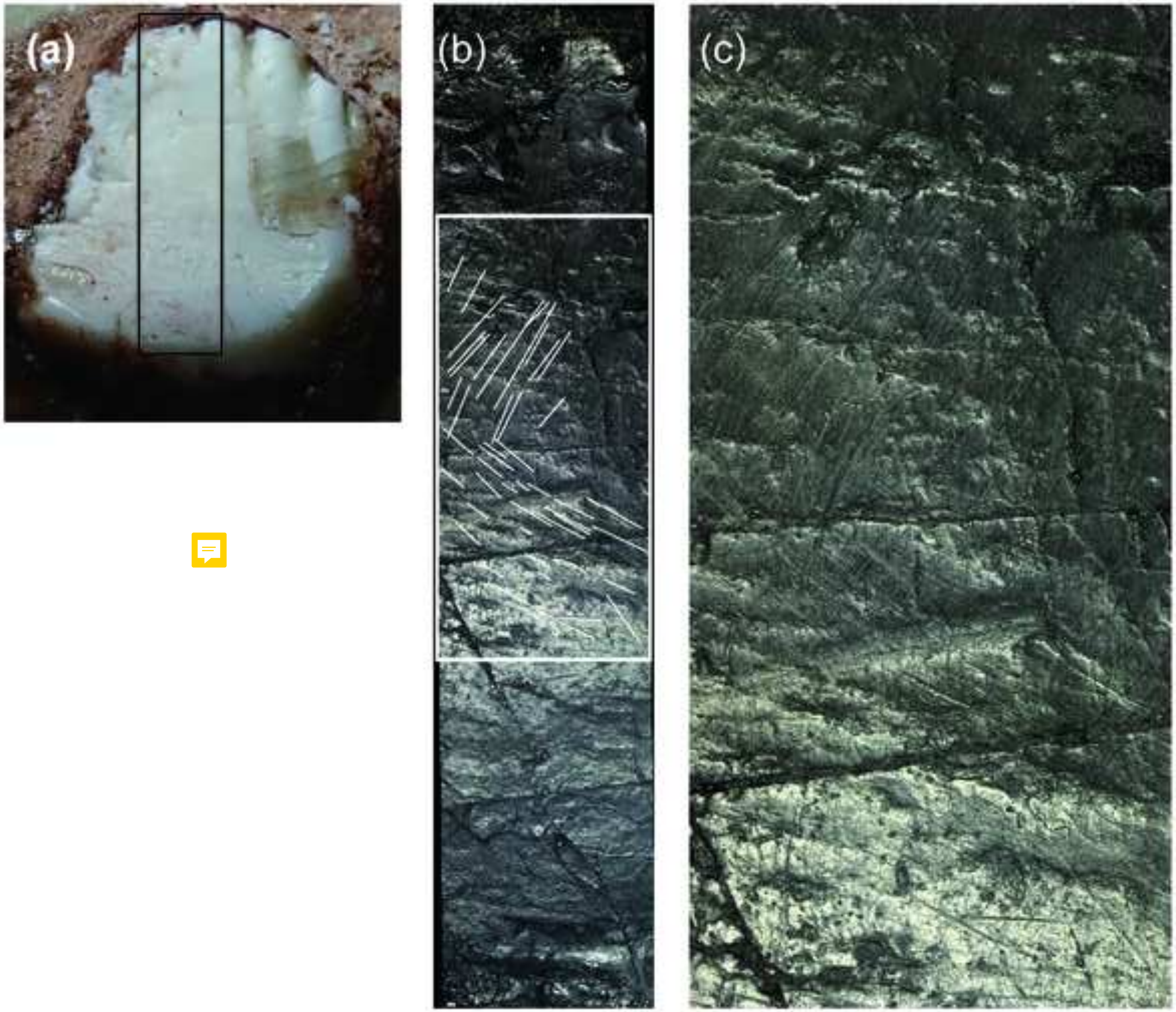

Figure 3

Click here to access/download;Figure;Fig 3 cs5.jpg

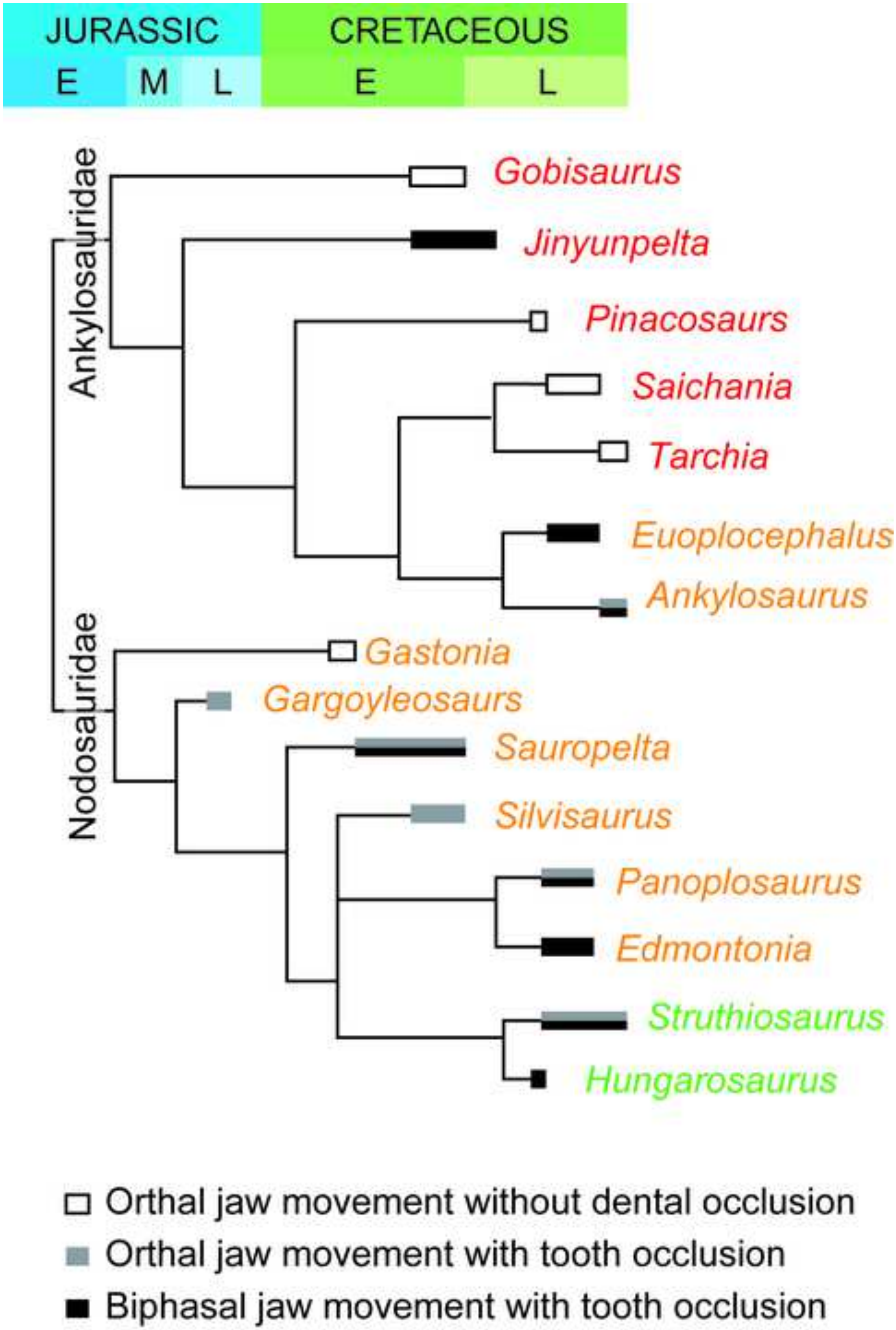

Supplement: Attachment — Submitted filename: PONE-D-20-40068_reviewer.pdf [file pone.0247969.s002.pdf]
